# Effect of fractioning on antibacterial activity of n-butanol fraction from *Enantia chlorantha* stem bark methanol extract

**DOI:** 10.1186/s12906-019-2459-y

**Published:** 2019-03-11

**Authors:** Rebeca Madeleine Ebelle Etame, Raymond Simplice Mouokeu, Frank Stève Monthe Poundeu, Igor Kenfack Voukeng, Cedric Laurel Pouaha Cidjeu, Alembert Tchinda Tiabou, Abel Joel Gbaweng Yaya, Rosalie Anne Ngono Ngane, Jules Roger Kuiate, François Xavier Etoa

**Affiliations:** 1 Institute of Medical Research and Medicinal Plant Studies, (IMPM), PO Box 13033, Yaoundé, Cameroon; 2 Institute of Fisheries and Aquatic Sciences, University of Douala, PO Box 7236, Douala, Cameroon; 3 Faculty of Sciences, University of Douala, PO Box 24157, Douala, Cameroon; 4 Faculty of Sciences, University of Dschang, PO Box 67, Dschang, Cameroon; 5 Faculty of Sciences, University of Yaoundé I, PO Box 812, Yaoundé, Cameroon

**Keywords:** *Enantia chlorantha*, Antibacterial activity, Palmitin

## Abstract

**Background:**

*Enantia chlorantha* is a plant belonging to Annonaceae Family. The Barks and leaves are used traditionally to treat infectious diseases. Earlier studies highlighted the antibacterial activity of stem barks methanol extract. This study is thus aimed at investigating the effect of fractionation on antibacterial activity of its n-butanol fraction.

**Methods:**

The extract of *E. chlorantha* stem barks was obtained by maceration in methanol and then subjected to a liquid/liquid partition by successive depletion with solvents of increasing polarity. The n-butanol fraction was fractionated by adsorption chromatography on silica gel. A product was isolated from the dichloromethane/methanol (2%) fraction and the structure was determined on the basis of spectroscopic data; Proton Nuclear Magnetic Resonance (^1^H NMR), *Carbon*-13 Nuclear *Magnetic Resonance* (^13^C NMR), Heteronuclear Multiple Bond Correlation (HMBC), H-correlation spectroscopy (H-COSY), attached proton test (APT), heteronuclear multiple quantum coherence (HSQC). The antibacterial activity was evaluated by broth microdilution method against six reference strains and eight clinical bacterial strains.

**Results:**

The n-butanol fraction was found to be active with MIC values ranging from 32 to 256 μg/mL. The F_A_ sub-fraction was more efficient among the eight sub-fractions, the n-butanol fraction and comparable to Chloramphenicol used as reference antibiotic. The product obtained was elucidated as palmitin. The antibacterial activity of the latter was comparable to that of Chloramphenicol on one reference strain and 4 of the 6 clinical strains.

**Conclusion:**

The F_A_ sub-fraction had better antibacterial activity than the n-butanol fraction and other sub-fractions, and possibly palmitin was the active substance responsible for the antibacterial activity of *E. chlorantha.*

## Background

The study of plant chemistry and its virtues for the treatment of diverse human diseases is very old. Notwithstanding, it is still important because the plant kingdom is a huge source of bioactive molecules [1]. Infectious diseases are among the top 10 causes of death and the leading cause of disability-adjusted life years worldwide. Amongst these, acute lower respiratory tract infections, diarrhoeal diseases and tuberculosis (TB) are responsible for significant global morbidity and mortality [2]. Despite the progress of antibiotic therapy, there is a constant increase of bacterial resistance, which is a brake on the control of bacterial infections and the cause of therapeutic failure [3]. It becomes important to find new substances that could overcome bacterial infections. This could be done through the valorization of ethnomedicinal preparations [4], improvement of local extraction techniques [5], search for pure active molecules from plant extracts [6], or search for more active extract fractions [7].

Among the several medicinal plants distributed worldwide, *E. chlorantha* (Annonaceae) barks and leaves are traditionally used for the treatment of urinary tract infections, malaria, and yellow fever [8]. Recent studies on this plant revealed antiviral [9], anti-cancer, antioxidant [10] and antimicrobial activities [11]. Previous studies from our research team highlighted the antibacterial activity of the stem barks methanol extract and a significant increase of this activity achieved with the n-butanol fraction following successive partition of this methanol extract [7]. As a continuation to those previous works, the current study was initiated to investigate the effect of further fractionation of the n-butanol fraction of *E. chorantha* stem bark methanol extract on its antibacterial activity.

## Methods

### Plant material

*E. chlorantha* barks were collected in April 2016 around the vicinity of Kalla Mountain located in Yaounde-Cameroun. The plant was authentified at the Cameroon National Herbarium in Yaoundé by Mr. Tadjouteu Fulber where a voucher specimen was deposited with the reference number 45569/HNC.

### Microorganisms

Fourteen bacteria strains including six reference strains and eight clinical strains were used for the experiment. These clinical strains were obtained from the ADLUCEM hospital at Bafang (*Escherichia coli*, *Enterobacter aerogenes*, *Klebsiella pneumoniae*, *Pseudomonas aeruginosa*) and the ‘Centre Pasteur’ of Cameroon (*Salmonella paratyphi A, Salmonella paratyphi B, Salmonella enteric* serovar *typhi*). Reference strains consisted of American Type Cell Culture (ATCC®). The characteristics of these bacteria had earlier been reported [7].

### Preparation of crude extract and fractions

*E. chlorantha* stem *barks* were dried at room temperature (24 ± 2 °C) for 30 days and ground into fine powder. The powdered material (1500 g) was macerated five times in 5 L of methanol for 72 h, and the filtrate obtained was concentrated under reduced pressure at 45 °C in a rotary evaporator to obtain the crude extract. The excess of methanol was eliminated by drying in an oven at 45 °C for 48 h and the extract was kept at 4 °C for future use.

For the partition, the methanol crude extract from *E. chlorantha* (80 g) was suspended in distilled water (300 mL) and extracted successively with different solvents including hexane, ethyl acetate and n-butanol. Dried fractions of this extract were obtained using rotary evaporator under reduced pressure at 45 °C [12].

### Fractionation of n-butanol fraction

Sixteen grams of n-butanol fraction from the partition were fixed on 32 g silica gel (250–300 Mesh) and latter introduced in the silica flash column (3 cm internal diameter and 50 cm high). The elution was done using solvent gradient (Table 1). Seventy two sub-fractions of 150 mL were collected and concentrated using rotary evaporator at 45 °C under reduced pressure, then mixed on the basis of their similarities on thin layer chromatography into eight sub-fractions labeled A-H.

**Table 1:** Yield of sub-fractions of the n-butanol fraction obtained from the methanol extract of *E. chlorantha* stem barks

Fractions	Solvent	Group	Weight (g)	Yield (%)
F_A_	Hex/DCM (50%)	F_1_-F_7_	0.094	0.58
F_B_	DCM/MeOH (2%)	F_8_-F_13_	5.4	33.75
F_C_	DCM/MeOH (2%)	F_14_-F_25_	4.32	20.75
F_D_	DCM/MeOH (2%)	F_26_-F_30_	1.41	8.19
F_E_	DCM/MeOH (4%)	F_31_-F_35_	0.12	0.75
F_F_	DCM/MeOH (4%) DCM/MeOH (6%)	F_36_-F_40_ F_41_	0.40	0.625
F_G_	DCM /MeOH (6%) DCM/MeOH (10%)	F_42_-F_56_ F_57_-F_60_	0.19	1.22
F_H_	DCM/MeOH (20%)	F_61_-F_72_	0.56	3.50

*Hex = Hexane, DCM = Dichloromethane, MeOH = Methanol, F = fractions A, B, C, D, E et F*

### Compound purification and structural analysis

F_B_ sub-fraction was obtained by grouped sub-fractions 8 to 13. Crystals were isolated from this sub-fraction by recrystallizing with hexane/ethyl acetate (*v*/v) followed by filtration. Hexane/ethyl acetate system (20%) was used to wash crystals and revelation was done with UV (254–350 μm) first and later by using sulfuric acid-EtOH (8):20). The compound obtained was labeled MF16.

The chemical structure of MF16 was elucidated using spectroscopic data such as RMN 1D (^1^H, ^13^C, APT) and RMN 2D (COSY, HMBC). RMN ^13^C data were set using HMQC experiments while fragment arrangements were done using COSY.

### Antibacterial activity

Bacterial suspensions of about 1.5 × 10^8^ CFU/mL (Mc Farland turbidity standard no. 0.5) were prepared 24 h old culture distilled water and diluted in Mueller Hinton broth culture medium (Liofilchem, Italy) to obtain a 1.5 × 10^6^ UFC/mL inoculum.

The in vitro antibacterial activity of n-butanol fraction, sub-fractions and isolated compound was performed by determining Minimum Inhibitory Concentrations (MIC) using broth microdilution methods in 96 wells microtiter plates [13]. The stock solution of n-butanol fraction, its sub-fractions and the purified compound were prepared in 2.5% dimethyl-sulfoxide (DMSO). Two-fold serial dilutions of the fraction or sub-fractions or pure substance were performed to obtain a final concentration ranging from 8 to 1024 μg/mL in a total volume of 100 μL/well. Bacterial suspension (100 μl) was seeded in wells to a final volume of 200 μL/well. Microplates were incubated at 37 °C for 24 h. Minimum Inhibitory Concentrations (MIC) were defined as the lowest concentration of extract required to prevent the color change of *p-* iodonitrotetrazolium chloride (INT); that is exhibited complete inhibition of bacterial cell growth [14]. Minimum Bactericidal Concentrations (MBC) were determined by sub-culturing 10 μL aliquot of the medium drawn from wells which did not show any growth after incubation during MIC assay and further incubated for 24 h at 37 °C for the appearance of colonies. The lowest concentration of the antibacterial agent from which negative growth was observed was considered as MBC [15]. The assays were carried out in triplicate and repeated twice. Chloramphenicol was used as positive control.

### Preliminary phytochemical screening

The n-butanol fraction and sub-fractions were screened for the presence of different classes of secondary metabolites including alkaloids, flavonoids, phenols, saponins, tannins, anthocyanins, quinones, sterols and triterpenes by chemical reaction methods using standard methods as previously described [16].

## Results

### Phytochemical screening

Phytochemical analysis of n-butanol fraction revealed the presence of phenols, tannins, flavonoids, quinones, triterpenes, alkaloids and sterols (Table 2). Phytochemical composition of sub-fractions revealed that F_F_ and F_G_ have the same composition (alkaloids, phenols and quinones) as well as F_D_ and F_E_ (alkaloids and phenols). Anthocyanins and saponins were absent while sterols present in n-butanol fraction could not be found in sub-fractions.

**Table 2:** Phytochemical composition of sub-fractions of n-butanol fraction obtained from the methanol extract of *E. chlorantha* stem barks

	Butanol	F_A_	F_B_	F_C_	F_D_	F_E_	F_F_	F_G_	F_H_
Alkaloids	+	+	+	+	+	+	+	+	+
Anthocyanins	–	–	–	–	–	–	–	–	–
Flavonoids	+	+	+	+	–	–	–	–	–
Phenols	+	–	+	+	+	+	+	+	+
Quinones	+	–	–	–	–	–	+	+	+
Saponins	–	–	–	–	–	–	–	–	–
Sterols	+	–	–	–	–	–	–	–	–
Tannins	+	–	–	–	–	–	–	–	+
Triterpenes	+	+	+	–	–	–	–	–	–

*+ = present; − = absent*

### Identification of isolated compound

The structure of MF16 compound was determined on the basis of spectral data. These structures were confirmed by comparing with those described in literature.

### Partial qualitative analysis

MF 16 crystallized into a yellowish-colored amorphous crystal and melted at 203–205. It was obtained in hexane/ethyl acetate (v: v) system, fluorescent with UV (254-350 μm). The pronounced green color of this compound during UV revelation suggested the presence of conjugated chromophores. It formed a precipitate with Mayer’s reagent suggesting that it is an alkaloid.

### Coupled analysis of the 1H, NMR Spectrum and 13C NMR Spectrum

The chemical structure of M16 was elucidated using physical and NMR data and compared with literature.

^1^H NMR (CD_3_OD, 500 MHz) δ 7.63 (1H, s, H-1), 7.04 (1H, s, H-4), 3.30 (2H, t, *J* = 6.3 Hz, H-5), 4.95 (2H, t, *J* = 6.3 Hz, H-6), 9.75 (1H, br- s, H-8), 8.09 (1H, d, *J* = 9.1 Hz, H-11), 8.01 (1H, d, *J* = 9.1 Hz, H-12), 8.79 (1H, s, H-13), 3.94 (3H, s, 2-OCH_3_), 4.00 (3H, s, 3-OCH_3_), 4.22 (3H, s, 9-OCH_3_), 4.10 (3H, s, 10-OCH_3_).

^13^C NMR (CD_3_OD, 500 MHz) δ 110.4 (d, C-1), 151.3 (s, C-2), 154.2 (s, C-3), 112.7 (d, C-4), 130.4 (s, C-4a), 28.2 (t, C-5), 56.4 (t, C-6), 146.7 (d, C-8), 123.6 (s, C-8a), 146.1 (s, C-9), 152.3 (d, C-10), 128.4 (d, C-11), 124.9 (d, C-12), 135.6 (s, C-12a), 121.7 (d, C-13), 140.1 (s, C-13a), 120.8 (s, C-13b), 57.5 (q, 2-OCH_3_), 57.1 (q, 3-OCH_3_), 63.0 (q, 9-OCH_3_), 57.8 (q, 10-OCH_3_).

Coupled analysis of the 1H-nuclear magnetic resonance (NMR) and 13C NMR spectra recorded in CD 3 OD at 500 and 125 MHz, revealed respectively the presence of signals characteristic of chemical shifts of protons and carbons of MF16.

The ^1^H NMR spectrum showed signals appearing as an AB system at δ_H_ 8.13 (1H, J = 10 Hz, H-11) and H 8.03 (^1^H, J = 10 Hz, H-12). The two carbon carrying these protons respectively resonated at δ_C_ 128, 4 (C-11) and 124, 9 (C-11) on the ^13^C NMR spectrum.

### HSQC/APT spectrum analysis

Coupled analysis of the HSQC/APT spectrum 135 revealed 10 negative signals (4 methoxy signals O-CH_3_–6 methine signals –CH) and 11 positive signals (9 quaternary carbon signals -C, 2 methylenes -CH_2_).

### Analysis of COSY and HMBC spectrum

The COZYCOSY spectrum of the aromatic zone of MF16 shows that it belongs to the berberin alkaloids class, revealing on one hand, two ^*3*^*J* correlations between the protons H5-H6 and H11-H12, and on the other, two ^*5*^*J* correlations between protons H1-H4 and H8-H13.

The HMBC spectrum enabled the positioning of the methoxy group due to ^*3*^*J* coupling between the methoxy protons and the carbons.

**Fig. 1:**
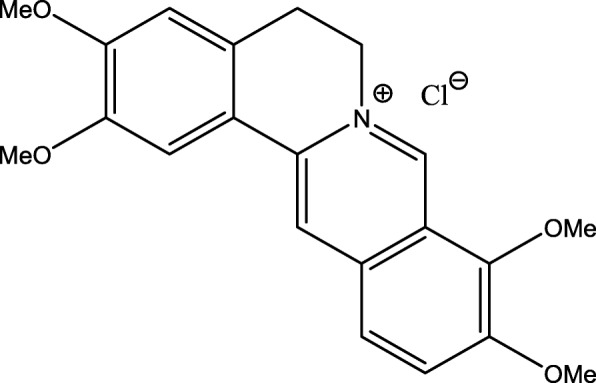
Chemical structure of Palmitin (MF16)

The set of physical and spectroscopic data compared with the literature made it possible to attribute to MF16 the following structure which is of palmitin (Fig. 1). It has a molecular weight of 352 g/mol, corresponding to the empirical formula C_21_H_22_NO_4_ + .

### Antibacterial activity

The MIC and MBC values and MBC/MIC ratio of n-butanol fraction from methanol extract of *E. chlorantha* stem barks and its sub-fractions against six reference ATCC® strains and eight clinical strains are presented in Table 5. This fraction inhibited bacteria growth with MIC values ranging from 32 to 256 μg/mL. It was found to be more efficient on *S. enterica serovar typhi* (SAL9), *E. coli* (E.C 136) and *S. aureus* (ST120). The MBC/MIC ratio showed bactericidal effect on 12 strains.

Sub-fractions of n-butanol fraction obtained from methanol extract of *E. chlorantha* stem barks were also tested for their antibacterial activity. Results showed that all of the eight sub-fractions were found to be effective against all the tested bacteria with MIC values ranging from 4 to 512 μg/mL (Tables 3 and 4). The F_A_ subfraction with MIC values ranging from 4 to 256 μg/mL was found to be more active when compared to other sub-fractions and n-butanol fraction with exception of *P. aeruginosa* (ATCC® 27,853™), where n-butanol fraction was more efficient with MIC value of 128 μg/mL. This F_A_ sub-fraction also showed significant activity on 7 bacteria strains (*E. aerogenes* ENT 119, *K. pneumoniae* (ATCC 11296, KL 128) *S. paratyphi* (SPA)*, S. aureus* (ATCC 25923), *S. aureus* (ST 120), *S. enteric serovar typhi* (SAL 9) compared to reference antibiotic Chloramphenicol.

**Table 3:** Minimum Inhibitory Concentrations (MIC), Minimum Bactericidal Concentrations (MBC) and MBC/MIC ratios (R) of n-butanol fraction and sub- fractions from the methanol extract of *E. chlorantha* stem barks (μg/mL)

Microorganisms		Butanol fraction			F_A_			F_B_			F_C_			F_D_			CHL		
Gram **-**		MIC	MBC	R	MIC	MBC	R	MIC	MBC	R	MIC	MBC	R	MIC	MBC	R	MIC	MBC	R
*E. coli*	ATCC 10536	128	256	2	8	128	16	64	256	4	128	> 512	4	256	> 512	–	4	64	16
	E.C 136	32	256	8	32	256	8	32	256	8	32	256	8	64	512	8	32	256	8
	E.C 137	64	256	4	64	512	8	64	256	4	64	256	4	64	512	8	64	> 512	–
	E.C 96	128	256	2	64	256	8	64	256	4	128	> 512	4	256	> 512	–	32	128	4
*E. aerogenes*	ATCC 13048	256	512	2	16	512	32	64	512	8	32	256	8	32	256	8	8	128	16
	ENT 119	64	512	4	8	128	16	32	256	8	32	512	8	128	512	4	16	128	8
*K. pneumoniae*	ATCC 11296	128	512	4	4	8	2	128	128	1	64	512	4	512	> 512	–	8	64	8
	KL 128	64	256	4	4	64	16	32	256	8	64	512	4	64	128	2	32	128	4
*S. paratyphi*	SPA	64	256	4	4	64	16	32	256	8	64	512	4	256	> 512	–	16	64	4
*S. typhi*	ATCC 6539	64	256	4	64	256	4	32	256	8	32	256	4	128	> 512	–	64	256	4
	SAL 9	32	512	16	16	256	8	32	> 512	–	128	> 512	16	128	> 512	–	32	128	4
*P. aeruginosa*	ATCC 27853	128	512	4	256	> 512	–	128	256	2	128	512	4	128	512	4	16	128	8
Gram **+**																			
*S. aureus*	ATCC 6539	256	512	2	4	32	8	32	256	8	64	128	2	128	> 512	4	32	128	4
	ST 120	32	512	4	4	64	16	32	256	8	16	256	4	64	512	8	8	64	8

**Table 4:** Minimum Inhibitory Concentrations (MIC), Minimum Bactericidal Concentrations (MBC) and MBC/MIC ratios (R) of n-butanol fraction and sub-fractions from the methanol extract of *E. chlorantha* stem barks (μg/mL)

		F_E_			F_F_			F_G_			F_H_			CHL		
Microorganisms		MIC	MBC	R	MIC	MBC	R	MIC	MBC	R	MIC	MBC	R	MIC	MBC	R
Gram **-**																
*E. coli*	ATCC 10536	128	> 512	–	128	512	4	64	> 512	–	256	512	2	4	64	16
	E.C 136	128	512	4	128	512	4	64	> 512	8	256	> 512	–	32	256	8
	E.C 137	128	> 512	–	256	512	2	128	512	4	64	> 512	–	64	> 256	–
	E.C 96	256	> 512	–	64	> 512	–	128	256	2	64	> 512	–	32	128	4
*E. aerogenes*	ATCC 13048	128	512	4	32	32	32	64	512	8	256	> 512	–	8	128	16
	ENT 119	128	256	2	64	128	16	64	512	8	256	> 512	–	16	128	8
*K .pneumoniae*	ATCC 11296	256	512	2	128	512	4	128	256	2	32	> 512	–	8	64	8
	KL 128	64	256	4	32	64	16	64	128	2	128	> 512	–	32	128	4
*S. paratyphi*	SPA	64	> 512	–	32	64	16	64	256	4	64	> 512	–	16	64-	4
*S. enterica* serovar *typhi*	ATCC6539	128	> 512	–	128	256	4	128	> 512	–	64	> 512	–	32	256	8
	SAL 9	256	> 512	–	64	256	8	64	512	8	256	> 512	–	32	128	4
*P. aeruginosa*	ATCC 27853	512	> 512	–	256	> 512	–	256	> 512	–	256	512	–	16	128	4
Gram***+***																
*S.aureus*	ATCC 6539	64	128	2	4	32	8	32	> 512	–	64	> 512	–	8	128	8
	ST 120	64	256	4	4	64	16	32	> 512	–	256	> 512	–	8	64	4

The isolated compound palmitin (MF16) revealed moderate activity against all isolates and strains tested with MIC values ranging between 16 to 64 μg/mL (Table 5). The more sensitive bacteria were *E. aerogenes* (ATCC 13048, ENT 119) and *K. pneumoniae* (KL128) with MIC value of 16 μg/mL.

**Table 5:** Minimum Inhibitory Concentrations (MIC), Minimum Bactericidal Concentrations (MBC) and MBC/MIC ratios (R) of palmitin (MF16) (μg/mL)

Microorganisms		MF 16			CHL		
		MIC	MBC	R	MIC	MBC	R
*E. coli*	ATCC 10536	64	256	4	4	64	16
	E.C 136	32	128	4	32	256	8
	E.C 137	32	128	4	64	> 256	–
	E.C 96	32	256	8	32	128	4
*E. aerogenes*	ATCC 13048	16	128	8	8	128	16
	ENT 119	16	256	16	16	128	8
*K. pneumoniae*	ATCC 11296	64	256	4	8	64	8
	KL 128	16	128	8	32	128	4
*S. aureus*	ATCC 25923	32	512	16	8	128	4
	ST 120	64	256	4	8	64	8

*CHL*: *Chloramphenicol*

## Discussion

Microbial infections continue to pose serious health problems in the world as a whole and in developing countries in particular. The use of plant extracts is nowadays essential in the Search for new active molecules against microbial agents [4]. They could act because of an active ingredient [6] or several active principles acting in synergy. In the latter case, it is interesting to find means to concentrate the metabolites responsible for optimal activity.

The n-butanol fraction of *E. chlorantha* stem bark has significant activity on all tested strains with MIC values ranging from 32 to 256 μg/ml. Previous work with the same plant showed MIC values with aqueous, methanol and ethanol extracts on clinical isolates ranged from 25 to 150 mg/mL [17]. Adesokan et al., [18] revealed the antibacterial activity of the aqueous bark extract of this plant on *S. aureus, B. subtilis, E. coli, P. aeruginosa, S. typhimurium* with MIC values between 25 and 105 mg/mL. These results are in line with those reported by our research team and point out the *E. clorantha* barks as a source of antibacterial compound. However, MIC values reported by those authors were higher. The differences could partly be attributed to the method used to investigate the antibacterial activity. Indeed, we refer to broth micro-dilution method to elucidate the antibacterial activity while the agar diffusion method was used by reported authors. Moreover, the parts of plant used as well as the extracted solvents could be additional factors which affected the antibacterial activity. These emphasize the necessity of standardized methods for the plants extraction as well as the evaluation of their antimicrobial activity.

According to several authors, the broth microdilution method appears to be more suitable for the evaluation of the activity of plants extract whose solubility is not quite known. It is also ideal for evaluating substances to be administered orally because the substance is in direct contact with microorganisms [19].

From the n-butanol fraction to sub-fractions FA, F_B_, FC and the product obtained, the antibacterial activity increases. This corroborates the idea that the active compound of the bark of this plant could be polar in nature as it can be deduced from this work. Furthermore, increase activity with fractionation reveals that the active principles of the stem barks of this plant are concentrated during fractionation in some fractions and highlights the fractionation as alternative to ameliorate plant extracts antimicrobial activity. Similar results were reported by several authors [7, 20].

F_A_ sub-fraction, obtained with hexane-dichloromethane system (v: v) showed strong antibacterial activity when compared to Chloramphenicol, used as a reference molecule. This fraction could therefore be used as active ingredient in the treatment of bacterial infections using *E. chlorantha* bark.

According to Kuete’s [21] criteria, palmitin revealed a significant antibacterial activity. Its activity was comparable to that of Chloramphenicol used as reference molecule on three bacterial strains. This suggests that this compound could be responsible for the antibacterial activity of *E. chlorantha* stem barks. This compound could therefore be used as marker for further studies to standardize substances from this plant in view of phytomedicine production. Such works are becoming more visible. Indeed, Dotsé et al., [22] made available a phytomedicine from the *Azadirachta indica* extract with the commercial name geduimine, a major compound isolated from this plant. The antibacterial activity of palmitin could be due to the presence of a benzopyridine nucleus within its structure. On this basis, it could be classified in the group of isoquinoline which acts on bacterial DNA by preventing its replication. Indeed, quinolones bind to the ends of the DNA strands, which can no longer connect. The formation of a DNA-isoquinolone complex is irreversible and leads to the death of the bacterial cell [23]. Palmitin could also serve as a backbone for the synthesis of new, more specific and more active molecules on bacteria. Similar work has been reported by Lemée [24].

The MBC/MIC ratios of sub-fractions and product were greater than or equal to 4 for most of the bacterial isolates and strains used. According to Djeussi et al. [15], the activity of these sub-fractions as well as palmitin could be bactericidal.

## Conclusion

The study was aimed at investigating the effect of fractionation of the n-butanol fraction of *E. chlorantha* methanol extract on the antibacterial activity. The F_A_ sub-fraction obtained with the dichloromethane/methanol system (50%) was found to be more active than Chloramphenicol used as reference antibiotic. Palmitin was isolated as metabolite responsible for this antibacterial activity.

The FA sub-fraction from *E. chlorantha* stem barks methanol extract could be used as the active ingredient in the treatment of bacterial infections.

## Abbreviations

13C NMR): Carbon-13 Nuclear Magnetic Resonance
1H NMR: Proton Nuclear Magnetic Resonance
APT: Attached proton test
ATCC: American Type Culture Collection
H-COSY: H-correlation spectroscopy
HMBC: Heteronuclear Multiple Bond Correlation
HSQC: Heteronuclear multiple quantum coherence
MBC: Minimum Bactericidal Concentrations
MIC: Minimum Inhibitory Concentrations
